# Dysregulation of cerebrospinal fluid metabolism profiles in spinal muscular atrophy patients: a case control study

**DOI:** 10.1186/s13052-024-01726-6

**Published:** 2024-08-22

**Authors:** Wei Zhuang, Minying Wang, Mei Lu, Zhehui Chen, Meifen Luo, Wanlong Lin, Xudong Wang

**Affiliations:** 1 Department of Pharmacy, Women and Children’s Hospital, School of Medicine, Xiamen University, Xiamen, China; 2 Department of Pediatrics, Women and Children’s Hospital, School of Medicine, Xiamen University, Xiamen, China; 3 Department of Xiamen Newborn Screening Center, Women and Children’s Hospital, School of Medicine, Xiamen University, Xiamen, China

**Keywords:** Spinal muscular atrophy, Metabolic disorder, Metabolomics, Cerebrospinal fluid, N-acetylneuraminic acid

## Abstract

**Background:**

Spinal muscular atrophy (SMA) is a neurodegenerative disorder. Although prior studies have investigated the metabolomes of SMA in various contexts, there is a gap in research on cerebrospinal fluid (CSF) metabolomics compared to healthy controls. CSF metabolomics can provide insights into central nervous system function and patient outcomes. This study aims to investigate CSF metabolite profiles in untreated SMA patients to enhance our understanding of SMA metabolic dysregulation.

**Methods:**

This case control study included 15 SMA patients and 14 control subjects. CSF samples were collected, and untargeted metabolomics was conducted to detect metabolites in SMA and control groups.

**Results:**

A total of 118 metabolites abundance were significantly changed between the SMA and control groups. Of those, 27 metabolites with variable importance for the projection (VIP) ≥ 1.5 were identified. The top 5 differential metabolites were N-acetylneuraminic acid (VIP = 2.38, Fold change = 0.43, *P* = 5.49 × 10^–5^), 2,3-dihydroxyindole (VIP = 2.33, Fold change = 0.39, *P* = 1.81 × 10^–4^), lumichrome (VIP = 2.30, Fold change = 0.48, *P* = 7.90 × 10^–5^), arachidic acid (VIP = 2.23, Fold change = 10.79, *P* = 6.50 × 10^–6^), and 10-hydroxydecanoic acid (VIP = 2.23, Fold change = 0.60, *P* = 1.44 × 10^–4^). Cluster analysis demonstrated that the differentially metabolites predominantly clustered within two main categories: protein and amino acid metabolism, and lipid metabolism.

**Conclusions:**

The findings highlight the complexity of SMA, with widespread effects on multiple metabolic pathways, particularly in amino acid and lipid metabolism. N-acetylneuraminic acid may be a potential treatment for functional improvement in SMA. The exact mechanisms and potential therapeutic targets associated with metabolic dysregulation in SMA require further investigation.

**Supplementary Information:**

The online version contains supplementary material available at 10.1186/s13052-024-01726-6.

## Background

Spinal muscular atrophy (SMA) is a genetic disease characterized by motor neuron dysfunction in the spinal cord, resulting in muscle weakness and atrophy [1]. The clinical manifestations of SMA vary widely, and patients are typically classified into five main groups (types 0–4) based on maximum motor function achieved [2, 3]. The most severe form is spinal muscular atrophy type 0, which leads to stillbirth, while untreated type 1 patients experience early infantile-onset progressive skeletal, bulbar, and respiratory muscle weakness, often leading to early mortality [1]. The underlying cause of SMA is pathogenic variants in the survival motor neuron 1 (*SMN1*) gene, which produces a protein essential for motor neuron survival [4]. The severity of the disease is inversely correlates with the number of copies of the *SMN2* [1]. In addition to affecting motor neurons, *SMN* pathogenic variants also affect various other systems such as skeletal muscle, heart, kidney, liver, pancreas, spleen, bone, connective tissues, and immune system thereby potentially impacting the metabolism of SMA patients [5, 6].

Despite the identification of *SMN1* pathogenic variant as the driving force behind SMA, the molecular mechanisms underlying the disease remain unclear. Metabolic studies are crucial for exploring biomarkers, revealing molecular features, and understanding the metabolic mechanisms involved [7]. Metabolomic analyses can help identify potential biomarkers for SMA, shedding light on disease progression and facilitating the development of novel therapies. Previous studies have shown huge changes in metabolomic profiles in SMA patients. For instance, serum creatinine has recently emerged as a potential biomarker for monitoring disease progression, with decreasing levels reflecting disease severity [8]. Compared to healthy individuals, SMA patients exhibit reduced numbers and concentrations of urine metabolites [6]. Notably, while several studies have explored the metabolomes of SMA patients in various contexts, there have been no reports on the CSF metabolomics of SMA patients compared to healthy controls. CSF metabolomics can provide insights into central nervous system function and serve as predictive and reflective biomarkers of patient outcomes [9].

Given these considerations, it is essential to compare the CSF metabolomic profiles of healthy controls and SMA patients. Such a comparison can help identify the metabolites involved in the cascade of biological events in SMA, explore potential therapeutic approaches, and identify biomarkers for the disease.

## Methods

### Patients and study design

This study follows a case–control design and includes a total of 15 SMA patients from the Women and Children's Hospital, School of Medicine, Xiamen University (China), as well as 14 control subjects. The enrollment period for this study was between December 1st, 2021, and September 30th, 2022. The inclusion criteria for the SMA group were as follows: individuals under 18 years old, confirmed SMA diagnosis through genetic testing, and receiving nusinersen treatment for the first time. Exclusion criteria included prior treatment with nusinersen, risdiplam, or onasemnogene abeparvovec, contraindications for nusinersen therapy, and contraindications for lumbar puncture. For the control group, the inclusion criteria were individuals under 18 years old with CSF samples collected because of first febrile seizures, while exclusion criteria included genetic metabolic diseases, those using medications such as antiepileptics (with the exception of acetaminophen), and inability to complete the follow-up management. To minimize confounding factors, all patients received the same management protocol. Prior to lumbar puncture, patients fasted for 6–8 h, and sample collection was performed between 8:00–9:00 a.m.

### Compliance with ethics

This study did not cause any additional harm to SMA patients or control subjects. All CSF samples were obtained from discarded samples. In the SMA group, to minimize the harm to patients, we only selected patients who were scheduled to receive nusinersen treatment, because the blank CSF is extracted in a volume equal to the administration volume before nusinersen injection. Therefore, we retained the discarded CSF samples before nusinersen injection. As for the control group, CSF samples were obtained from febrile seizures patients. However, only samples from confirmed non-encephalitis cases with a 3-month follow-up indicating good health were included in this study.

This study received approval from the ethical committee of the Women and Children's Hospital, School of Medicine, Xiamen University (IEC-XJS-2021–04). Written informed consent was obtained from all participating subjects’ legal guardians.

### Sample collection

Approximately 2 mL of CSF was collected and immediately centrifuged at 4°C at 14,000 rpm for 10 min. The supernatant was collected and frozen at -80°C until further analysis.

### Sample pretreatment

To extract total metabolites, sample pretreatment was conducted as follows: 400 µL of pre-cooled methanol was added to 100 µL of CSF samples, followed by vortexing. The mixture was then centrifuged, and all the supernatant was transferred and concentrated in a vacuum. The samples were subsequently dissolved in 150 µL of an 80% methanol solution containing 2-chlorobenzalanine (4 ppm), and the supernatant was filtered using a 0.22 μm membrane. Quality control samples were prepared by taking 20 µL from each sample. These samples were used for liquid chromatography-mass spectrometry (LC–MS) detection.

### Metabolomics detection

Metabolomics detection was performed using a Thermo Ultimate 3000 system equipped with an ACQUITY UPLC® HSS T3 column (150 × 2.1 mm, 1.8 µm, Waters) maintained at 40℃ for chromatographic separation. Analytes were eluted using a gradient elution method. For LC-ESI ( +)-MS analysis, the mobile phases consisted of a mixture of 0.1% formic acid in acetonitrile (v/v) (B2) and 0.1% formic acid in water (v/v) (A2). The separation process followed a gradient elution program as follows: 0 ~ 1 min, 2% B2; 1 ~ 9 min, 2% ~ 50% B2; 9 ~ 12 min, 50% ~ 98% B2; 12 ~ 13.5 min, 98% B2; 13.5 ~ 14 min, 98% ~ 2% B2; 14 ~ 20 min, 2% B2. For LC-ESI (-)-MS analysis, the mobile phases included acetonitrile (B3) and ammonium formate (5 mM) in water (A3). The separation conditions were as follows: 0 ~ 1 min, 2% B3; 1 ~ 9 min, 2% ~ 50% B3; 9 ~ 12 min, 50% ~ 98% B3; 12 ~ 13.5 min, 98% B3; 13.5 ~ 14 min, 98% ~ 2% B3; 14 ~ 17 min, 2% B3. Prior to injection into the LC–MS/MS system, each sample was assigned a random number.

The LC–MS assay was performed using a Thermo Q Exactive mass spectrometer. In LC-ESI ( +)-MS and LC-ESI (-)-MS modes, the spray voltages were set at 3.5 kV and -2.5 kV, respectively, with a capillary temperature of 325 ℃ and a normalized collision energy of 30 eV. The mass spectrometer conducted a full scan over the m/z range of 100–1,000 with a mass resolution of 70,000.

### Metabolomics data analysis and quality control

#### Data preprocessing

Raw data was converted into the mzXML format using Proteowizard software (v3.0.8789). Peak identification, filtration, and alignment were carried out using the XCMS package in R (v.3.3.2). This process allowed for the acquisition of the mass-to-charge ratio (m/z), retention time, and relative ratio of the peak area.

#### Quality control and quality assurance [10, 11]

A quality control sample was prepared by mixing 20 µL from each individual sample. These quality control samples were utilized to monitor any deviations in the analytical results compared to the pool mixtures and to assess potential errors generated by the analytical instrument itself. Peaks with a relative standard deviation ≤ 30% were retained for subsequent analyses to ensure data quality.

### Bioinformatics analysis

#### Hierarchical clustering

Hierarchical clustering was employed in this study using the agglomerative approach. Relative quantitative levels of metabolites were determined using the Pheatmap package in R 3.3.2. Samples and related data were clustered using the average-linkage clustering method based on a distance matrix.

#### Multivariate analysis

To show the difference metabolites profile of each group, multivariate analysis was performed after preprocessing of the data with autoscaling, mean-centering, and scaling to unit variance. Principal component analysis (PCA), partial least squares-discriminant analysis (PLS-DA), and orthogonal partial least squares discriminant analysis (OPLS-DA) were conducted using SIMCA-P v.13.0 and the ropls package in R.

#### Identification of differential abundant metabolites

To discover the contributable variables for classification, we applied the *P* value, Variable Importance Projection (VIP) produced by OPLS-DA, and fold change (FC). We considered metabolites to be statistically significant if their *P* value was less than 0.05 and their VIP values were greater than 1. Precise molecular weight of metabolites was first confirmed (molecular weight error < 30 ppm) followed by annotation and acquisition of corresponding information through Metlin (http://metlin.scripps.edu), MoNA (https://mona.fiehnlab.ucdavis.edu), and a standard database built by BioNovoGene Co., Ltd. (Suzhou, China) according to MS/MS fragmentation patterns. Normalization into relative content on the same level was done using a metabolite database built by BioNovoGene Co., Ltd. Agglomerate hierarchical clustering was conducted and differential metabolites were displayed via a heat map. Statistical analysis of differential metabolites was depicted in a volcano plot. Metabolic pathway analysis was based on Kyoto Encyclopedia of Genes and Genomes (KEGG) and referred to hypergeometric test [12].

### Statistical analysis

All statistical analyses were performed using IBM SPSS version 21.0. Categorical variables were analyzed using either the χ^2^ test or Fisher’s exact test, while continuous variables were analyzed using the Mann–Whitney U-test to compare two subgroups. Correction for multiple comparisons was performed using the Benjamini–Hochberg procedure. Statistical significance was assumed for *P* values less than 0.05 in untargeted metabolomics results.

## Results

### Patients’ characteristics

A total of 15 patients with SMA and 14 control subjects were included in this study (Table 1). Among the SMA group, 40% were girls, while in the control group, 64% were girls. The mean age of the SMA group was 2.3 years, and the control group had a mean age of 1.8 years. The mean height of the SMA group was 1.25 m, whereas the control group had a mean height of 0.80 m. The mean weight of the SMA group was 29.1 kg, and the control group had a mean weight of 11.0 kg. In the SMA group, there was one patient classified as type 1, nine patients as type 2, and five patients as type 3. Regarding the detection of *SMN1* and *SMN2*, all patients exhibited *SMN1* exon 7 homozygous deletion, and among them, five patients had 2 *SMN2* copies, eight patients had 3 *SMN2* copies, and two patients had 4 *SMN2* copies.

**Table 1:** Patients and control subjects characteristics

Characteristics	SMA	Control
Gender (n, girl/boy)	6/9	9/5
Age (year, mean ± SD)	2.3 ± 2.8	1.8 ± 2.9
Height (m, mean ± SD)	1.25 ± 0.3	0.80 ± 0.3
Weight (Kg, mean ± SD)	29.1 ± 17.0	11.0 ± 7.8
SMA type (n, 1/2/3)	1/9/5	/
*SMN1* pathogenic variant (n, exon7)	15	/
*SMN2* copy number (n, 2/3/4)	5/8/2	/

### Multivariate statistical analysis of metabolites

**Fig. 1:**
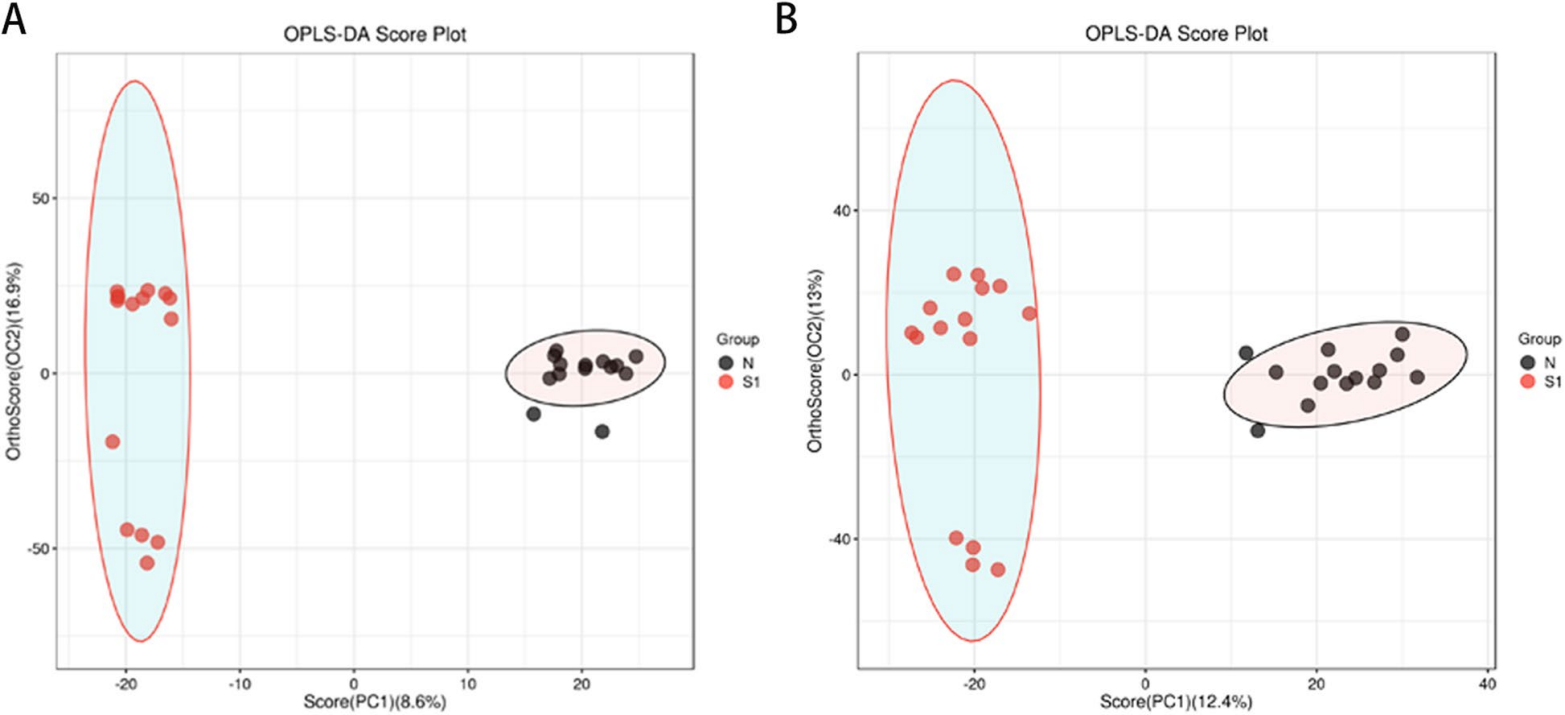
OPLS-DA models to separate patients with SMA from controls. **A** OPLS-DA plot for the positive ion model; **B** OPLS-DA plot for the negative ion model

Multivariate statistical analysis, including supervised methods such as OPLS-DA (Fig. 1) and PLS-DA (Additional file 1), as well as unsupervised analysis using PCA (Additional file 1), demonstrated clear differentiation between all SMA patients and normal controls in both positive and negative ionization modes. These findings indicate a significant metabolic profile alteration in the CSF of SMA patients.

### Differentially abundant metabolites in SMA group compared with normal group

**Fig. 2:**
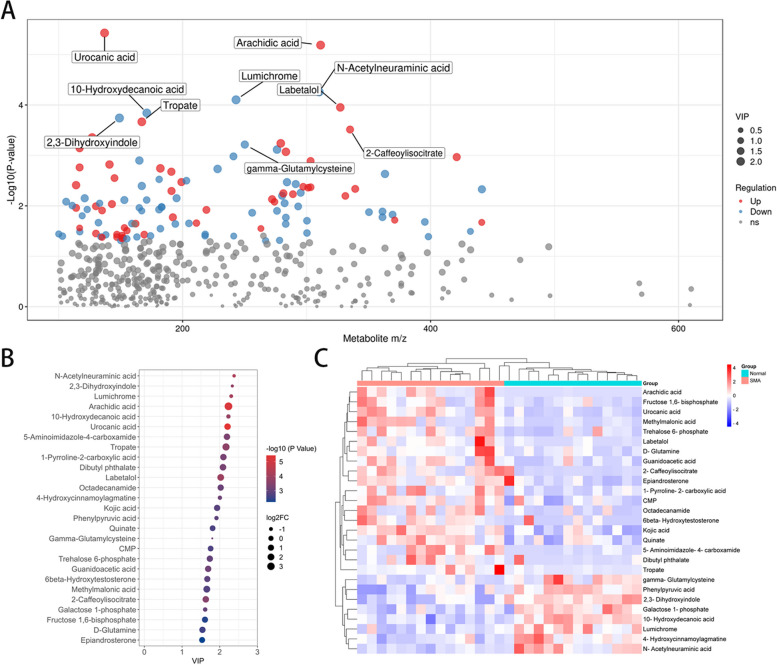
Changes of major metabolites in cerebrospinal fluid samples from SMA group and control group. **A** The fold change, VIP and *P* value of differentially abundant metabolites; **B** Heatmap clustering of differentially abundant metabolites in SMA group and control group; **C** Differences in metabolic material charge ratio and *P* value scatter plot

**Fig. 3:**
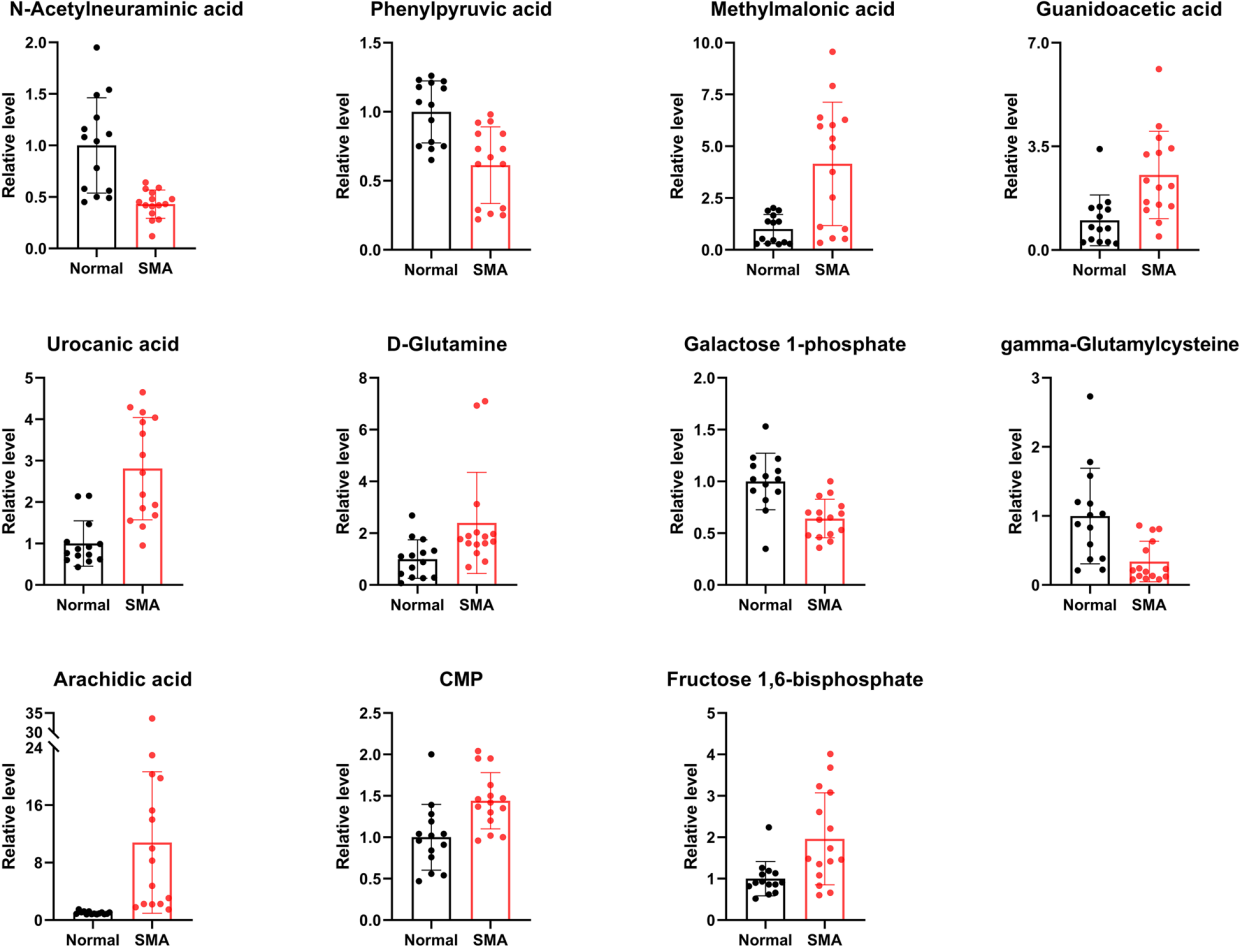
Differentially abundant metabolites in cerebrospinal fluid samples from the SMA group and the control group

A total of 118 metabolites level were found to be significantly changed between the SMA and control groups, with 52 exhibiting upregulation and 66 downregulation, the distribution of m/z and *P*-value was shown in Fig. 3C. Of these, 27 metabolites with VIP scores of 1.5 or greater were identified (Fig. 2A, Table 2), and cluster analysis was performed (Fig. 2B). The top 5 differential metabolites were N-acetylneuraminic acid (VIP = 2.38, Fold change = 0.43, *P* = 5.49 × 10^–5^), 2,3-dihydroxyindole (VIP = 2.33, Fold change = 0.39, *P* = 1.81 × 10^–4^), lumichrome (VIP = 2.30, Fold change = 0.48, *P* = 7.90 × 10^–5^), arachidic acid (VIP = 2.23, Fold change = 10.79, *P* = 6.50 × 10^–6^), and 10-hydroxydecanoic acid (VIP = 2.23, Fold change = 0.6, *P* = 1.44 × 10^–4^). A selection of the differentially abundant metabolites is shown in Fig. 3.

**Table 2:** Differentially abundant metabolites in SMA group compared with normal group

Name	Fold Change	VIP	*P*.value	FDR
N-Acetylneuraminic acid	0.43	2.38	5.49E-05	0.011
2,3-Dihydroxyindole	0.39	2.33	1.81E-04	0.020
Lumichrome	0.48	2.30	7.90E-05	0.012
Arachidic acid	10.79	2.23	6.50E-06	< 0.001
10-Hydroxydecanoic acid	0.6	2.23	1.44E-04	0.017
Urocanic acid	2.81	2.21	3.74E-06	< 0.001
5-Aminoimidazole-4-carboxamide	2.72	2.19	4.43E-04	0.029
Tropate	5.7	2.17	2.16E-04	0.021
1-Pyrroline-2-carboxylic acid	2.78	2.10	5.45E-04	0.031
Dibutyl phthalate	3.09	2.09	5.76E-04	0.031
Labetalol	3.7	2.03	1.12E-04	0.004
Octadecanamide	2.41	2.03	8.49E-04	0.036
4-Hydroxycinnamoylagmatine	0.59	2.00	7.66E-04	0.034
Kojic acid	2.26	1.93	1.52E-03	0.018
Phenylpyruvic acid	0.61	1.91	1.26E-03	0.046
Quinate	1.47	1.81	2.11E-03	0.022
gamma-Glutamylcysteine	0.34	1.80	6.10E-04	0.011
CMP	1.44	1.76	1.93E-03	0.021
Trehalose 6-phosphate	2.48	1.74	1.08E-03	0.015
Guanidoacetic acid	2.53	1.69	7.21E-04	0.011
6beta-Hydroxytestosterone	2.06	1.67	1.29E-03	0.016
Methylmalonic acid	4.15	1.66	1.73E-03	0.020
2-Caffeoylisocitrate	3	1.63	3.07E-04	0.007
Galactose 1-phosphate	0.64	1.61	1.05E-03	0.014
Fructose 1,6-bisphosphate	1.96	1.61	4.61E-03	0.035
D-Glutamine	2.4	1.54	2.81E-03	0.026
Epiandrosterone	1.71	1.53	5.87E-03	0.042

*VIP* Variable importance for the projection, *FDR* False Discovery Rate

Among the differential metabolites, 11 were related to amino acid metabolism. Gamma-glutamylcysteine (VIP = 1.80, Fold change = 0.34, *P* = 5.49 × 10^–5^), 2,3-dihydroxyindole (VIP = 2.33, Fold change = 0.39, *P* = 1.81 × 10^–4^), 4-hydroxycinnamoylagmatine (VIP = 2.00, Fold change = 0.59, *P* = 7.66 × 10^–4^), and phenylpyruvic acid (VIP = 1.91, Fold change = 0.61, *P* = 1.26 × 10^–3^) were found to have lower levels in the SMA group, while methylmalonic acid (VIP = 1.66, Fold change = 4.15, *P* = 1.73 × 10^–3^), urocanic acid (VIP = 2.21, Fold change = 2.81, *P* = 3.74 × 10^–6^), 1-pyrroline-2-carboxylic acid (VIP = 2.10, Fold change = 2.78, *P* = 5.45 × 10^–4^), D-glutamine (VIP = 1.54, Fold change = 2.40, *P* = 2.81 × 10^–3^), guanidoacetic acid (VIP = 1.69, Fold change = 2.53, *P* = 7.21 × 10^–4^), fructose 1,6-bisphosphate (VIP = 1.61, Fold change = 1.96, *P* = 4.61 × 10^–3^), and quinate (VIP = 1.81, Fold change = 1.47, *P* = 2.11 × 10^–3^) exhibited higher levels in the SMA group.

Moreover, three metabolites related to lipid metabolism were also found to be significantly differentially abundant: arachidic acid (VIP = 2.23, Fold change = 10.79, *P* = 6.50 × 10^–5^), epiandrosterone (VIP = 1.53, Fold change = 1.71, *P* = 5.87 × 10^–3^), and galactose 1-phosphate (VIP = 1.61, Fold change = 0.64, *P* = 1.05 × 10^–3^).

### Functional pathway analysis of differentially abundant metabolites

**Fig. 4:**
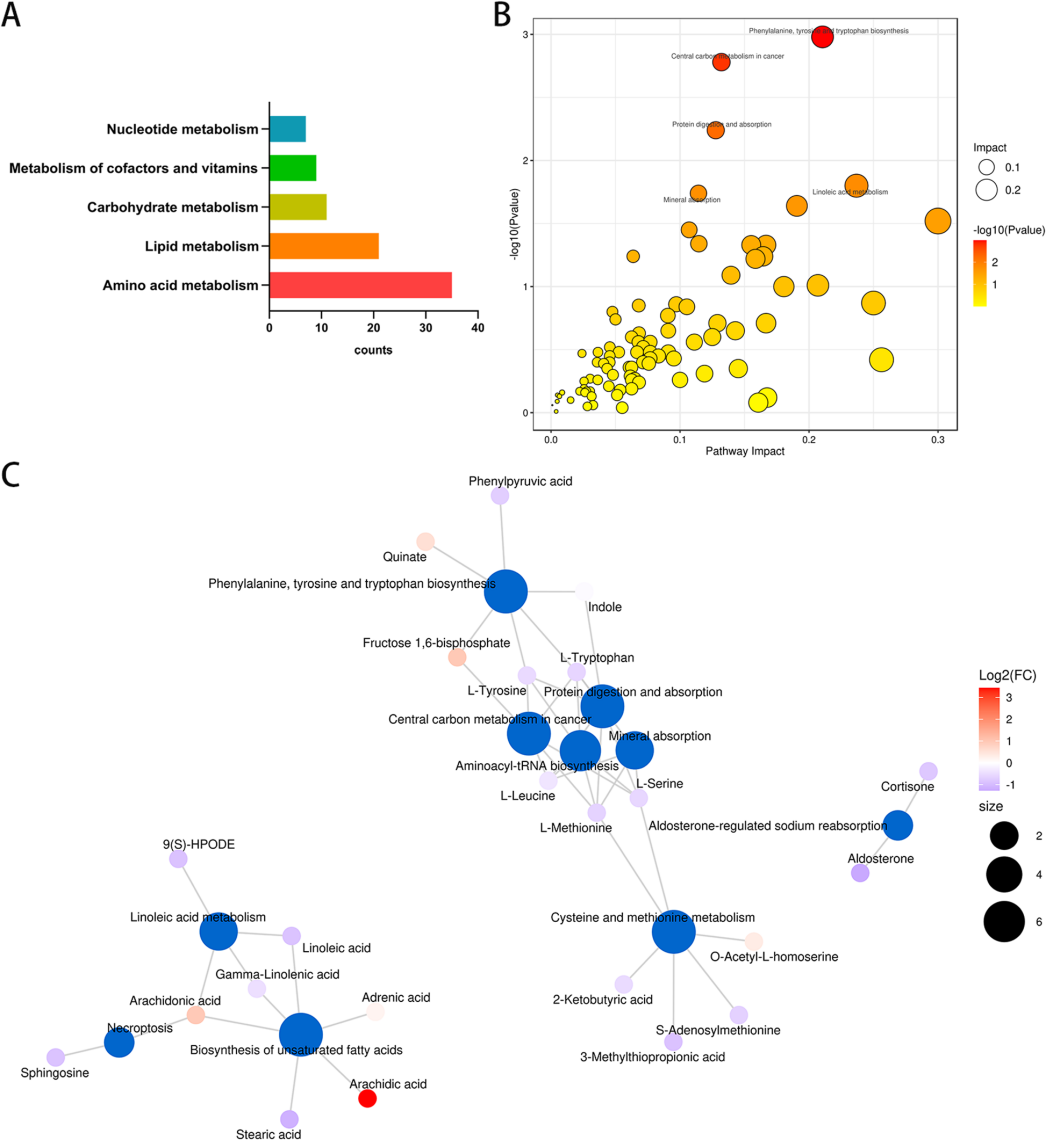
Pathway analysis of differentially abundant metabolites. **A** Histogram of top five pathways. The comparison between the control group and the SMA group showed the most significant changes in amino acid and lipid metabolism pathway; **B** Bubble diagram of pathways; **C **Network diagram

Among the 118 significant difference metabolites, a comprehensive analysis revealed that 35 metabolites were involved in amino acid metabolism, 21 in lipid metabolism, 11 in carbohydrate metabolism, 9 in cofactors and vitamins metabolism, and 7 in nucleotide metabolism (Fig. 4A).

To gain insights into the disrupted metabolic pathways in SMA, we conducted KEGG analysis (Fig. 4B). Notably, the top three pathways identified were phenylalanine, tyrosine, and tryptophan biosynthesis, central carbon metabolism, and protein digestion and absorption. These findings highlight the potential dysregulation of these pathways in SMA. Furthermore, cluster analysis (Fig. 4C) demonstrated that the differentially abundant metabolites predominantly clustered within three main categories: protein and amino acid metabolism, lipid metabolism, and aldosterone-regulated sodium reabsorption.

## Discussion

Understanding the biochemical changes of SMA patients is critical to understand the disease mechanism. To the best of our knowledge, this is the first study to investigate the alteration of CSF metabolism profiles in untreated SMA patients compared with control.

The identification and characterization of differentially abundant metabolites in CSF of SMA provide valuable insights into the underlying metabolic dysregulation in this disease. Our analysis revealed significant alterations in amino acid, lipid, carbohydrate, cofactors and vitamins, and nucleotide metabolism. The metabolites profiles of plasma [13, 14] and urine [6] in SMAs also showed change. The alteration of metabolomics may be a result of SMN protein deficiency or indirectly caused by nutritional deficiencies or inadequate intake, ultimately affecting cell growth and human body functions. These findings suggest that multiple metabolic pathways are affected in SMA, highlighting the complex nature of the disease.

### Amino acid metabolism disorder

The dysregulation of amino acid metabolism observed in SMA is of particular interest. Several key metabolites involved in this pathway, such as such as methylmalonic acid, and glutamine exhibited higher levels in the SMA group, indicating potential disruptions in amino acid utilization and clearance.

Increased levels of plasma methylmalonic acid can lead to methylmalonic acidemia, which can cause multi-organ damage including the brain, liver, and kidneys [15, 16]. The mechanism of damage may be related to mitochondrial dysfunction and neuronal apoptosis [15, 16]. Interestingly, there has no reports on methylmalonic acid abnormalities reported in SMA yet. Elevated levels of methylmalonic acid in cerebrospinal fluid may poisonous to the nervous system that affects the progression or severity of SMA. Some symptoms observed in SMA patients are similar to those seen in methylmalonic acidemia, such as seizures, decreased muscle tone, and poor feeding [15]. Vitamin B12 is a useful supplement for methylmalonic acidemia patients [17], and it may be a potential medicine for high methylmalonic acid SMA patients.

Meanwhile, glutamine is the most abundant and versatile amino acid in the body [18], is the main source of excitatory neurotransmitter, glutamate [19–21]. Alterations to glutamine metabolism appears to play a particularly key role in neurodegenerative diseases [22]. It has also been suggested in SMA autopsy that oxidative stress and glutamate transport may be partially involved in motor neuron destruction in patients with type 1 SMA [23]. However, the elevated levels of glutamine are associated with abnormalities in amino acid metabolism, ammonia metabolism, and urinary acid metabolism [18, 24]. There was a study reported that long-term glutamine supplement will lead to abnormalities in aminoacidemia-increased plasma levels of glutamine, glutamate, citrulline, ornithine, arginine, and histidine and decreased levels of valine, leucine, isoleucine, glycine, threonine, serine, and proline [25]. These results were coincidence with our study in CSF. There was a study reported that the decease level of glutamate and aspartate [26]. Additionally, there was a study reported that nusinersen treatment showed a significant regulating effect in amino acid metabolism [27].This study supported that the importance of amino acid metabolism in SMA.

However, the control group in this study included subjects with bacterial and viral infections, which could potentially influence amino acid metabolism and introduce bias [28–30]. Therefore, further confirmation of methylmalonic acid or glutamine level in cerebrospinal fluid through larger samples is required. Future studies should clarify the impact of methylmalonic acid and glutamine in cerebrospinal fluid on the onset and progression of SMA, as well as explore the feasibility of vitamin B12 supplementation as an adjunctive therapy.

### Lipid metabolism disorder

Our analysis also revealed marked changes in lipid metabolism in SMA. Arachidic acid, epiandrosterone, and galactose 1-phosphate were identified as differentially abundant metabolites in this pathway. These findings suggest that fatty acid metabolism and lipid signaling processes are impaired in SMA. The abnormal lipid metabolism is consistent with other reports [13, 27, 31, 32]. Previous studies have shown that patients with SMA may have profound malnutrition and dysmetabolism issues [31], especially lipid metabolic disorders, and these are currently considered to be the most significant metabolic issue in these patients. Dyslipidemia has been found in a large number of SMA patients, and abnormal fatty acid metabolism has also been found in *smn2B*/ -mice, with increased free fatty acids, total cholesterol, diacylglycerol, and hepatic triglyceride [32]. Patients with SMA are prone to dyslipidemia, hepatic steatosis, and non-alcoholic fatty liver disease [31, 32].

Arachidic acid, is an important fatty acid in biological systems, high levels of arachidic acid have been observed in individuals with certain metabolic disorders, such as adrenoleukodystrophy [33]. High levels of galactose 1-phosphate are commonly seen in patients with galactosemia patients [34]. Although SMA and galactosemia are both genetic disorders, they have different clinical manifestations and affect different systems in the body. Further investigations are warranted to explore the functional implications of these lipid metabolism alterations in the pathogenesis of SMA.

### Carbohydrate, cofactors and vitamins metabolism disorder

In addition to amino acid and lipid metabolism, disruptions in carbohydrate metabolism, as well as cofactors and vitamins metabolism, has been observed in SMA. The involvement of these pathways further highlights the complexity of the CSF microenvironment and metabolic dysregulation in SMA. While the direct link between SMA and carbohydrate metabolism is not well understood, it is possible that the disruption of SMN protein function may indirectly affect various metabolic processes, including carbohydrate metabolism or cofactors and vitamins metabolism. The identification of these dysregulated pathways provides valuable starting points for future research aimed at unraveling the underlying mechanisms and potential therapeutic targets in SMA. However, the exact mechanisms and implications are still under investigation.

### Others

We found that the CSF level of N-acetylneuraminic acid was lower than that of the control group. N-acetylneuraminic acid, also known as sialic acid, is involved in immunology and tumor biology [35]. The mechanism of the decrease of N-acetylneuraminic acid was unclear. N-acetylneuraminic acid cannot be synthesized by human [35]. Therefore, the low level of N-acetylneuraminic acid might due to the low intake. N-acetylneuraminic acid rescued pharyngeal pumping rate and egg-laying in *SMN1* phenotypic dysfunction *Caenorhabditis elegans* SMA model [36]. N-acetylneuraminic acid maybe a potential treatment to improve function in SMA patients. Further molecular and clinical studies should be performed to confirm the effects of N-acetylneuraminic acid in SMA.

### Limitations

This study has several limitations. First, it is important to acknowledge the limitations of this study. The sample size used in our analysis was relatively small, and further validation studies with larger cohorts are needed to confirm our findings. Second, the control group we enrolled was not a completely healthy control group. Subjects in these control groups received acetaminophen for antipyretic treatment, which may affect the metabolism of CSF prostaglandins [37, 38]. Additionally, the presence of bacterial or viral infections could influence CSF metabolites, potentially introducing bias [28–30, 39, 40]. However, all participants who provided CSF were confirmed to be healthy individuals during long-term follow-up. Third, the cross-sectional design of our study constrains our ability to establish causal relationships between differentially abundant metabolites and SMA. Longitudinal studies, which track changes over time, could provide more definitive evidence of causality and yield more robust conclusions than those drawn from cross-sectional analyses. Fourth, it is necessary to validate the differential metabolites at the animal level to strengthen our conclusions. Additionally, this approach could facilitate the exploration of new drug targets.

## Conclusions

In conclusion, our study provides new insights into the metabolic perturbations associated with SMA. The dysregulation of amino acid, lipid, carbohydrate, cofactors and vitamins, and nucleotide metabolism indicates a global disruption of metabolic homeostasis in SMA patients, especially amino acid, and lipid metabolism. In addition, N-acetylneuraminic acid may be a potential treatment for functional improvement in SMA patients. Further investigations are needed to fully understand the functional implications of these alterations and their potential as therapeutic targets in SMA.

### Supplementary Information

Supplementary Material 1.

## Data Availability

The datasets used and/or analysed during the current study are available from the corresponding author on reasonable request.

## Abbreviations

CSF: Cerebrospinal fluid
FC: Fold change
KEGG: Kyoto Encyclopedia of Genes and Genomes
LC-MS: Liquid chromatography-mass spectrometry
OPLS-DA: Orthogonal partial least squares discriminant analysis
PCA: Principal component analysis
PLS-DA: Partial least squares-discriminant analysis
SMA: Spinal muscular atrophy
SMN: Survival motor neuron
VIP: Variable importance for the projection
