# Supplementary material for: Dysregulation of cerebrospinal fluid metabolism profiles in spinal muscular atrophy patients: a case control study
Source: Ital J Pediatr. 2024 Aug 22;50:154. doi: 10.1186/s13052-024-01726-6 (PMC11342544; doi:10.1186/s13052-024-01726-6)
Supplement: Supplementary file 1 — Supplementary Material 1. [file 13052_2024_1726_MOESM1_ESM.docx]

**
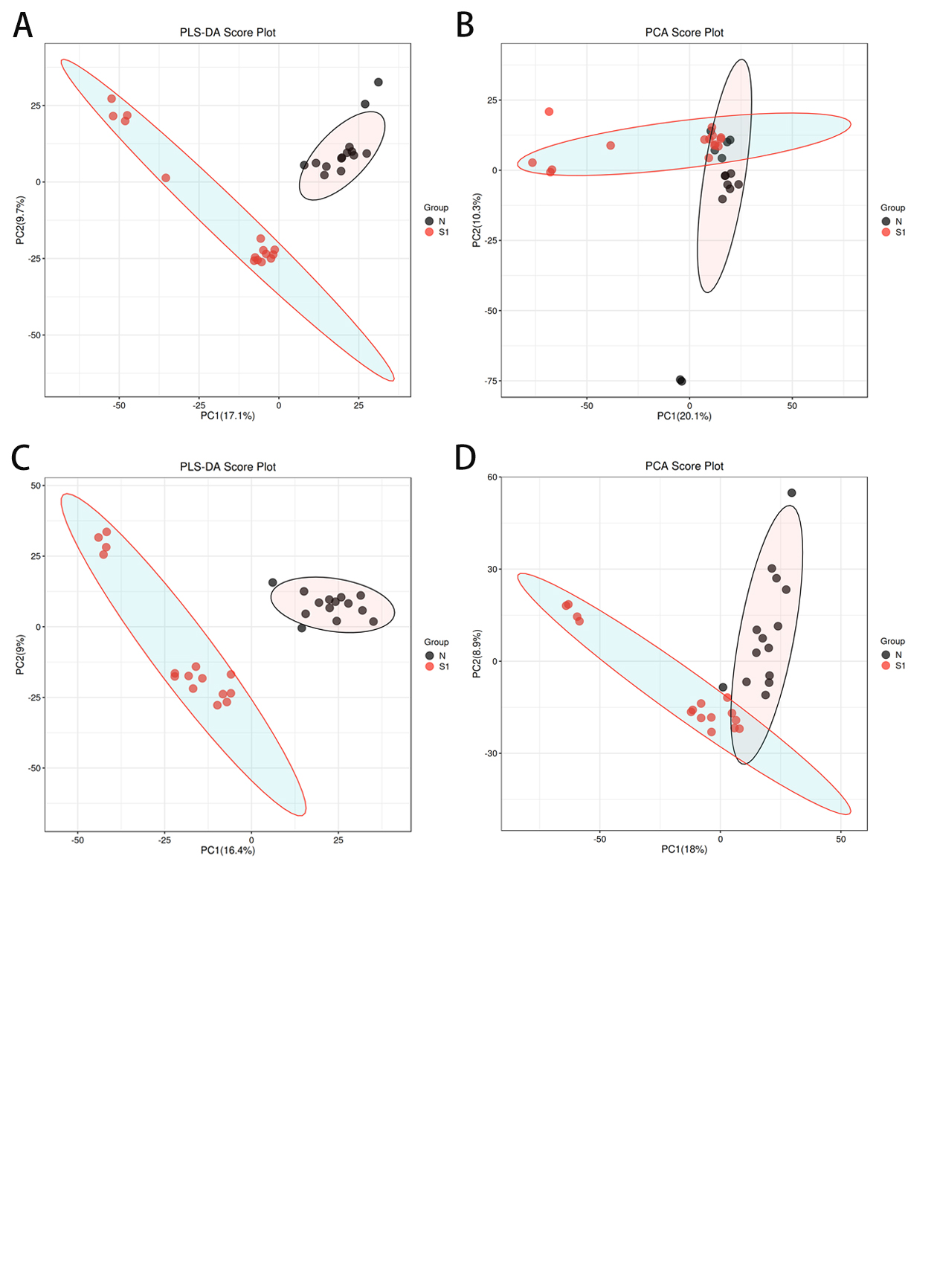
**

**Additional file 1** PLS-DA models to separate patients with SMA from controls. (**A**) PLS-DA plot for the positive ion model; (**B**) PLS-DA plot for the negative ion model.
